# Analysis of risk and predictors of brain radiation necrosis after radiosurgery

**DOI:** 10.18632/oncotarget.6532

**Published:** 2015-12-10

**Authors:** Hongqing Zhuang, Yi Zheng, Junjie Wang, Joe Y. Chang, Xiaoguang Wang, Zhiyong Yuan, Ping Wang

**Affiliations:** ^1^ Department of Radiotherapy, Tianjin Medical University Cancer Institute and Hospital, National Clinical Research Center for Cancer, Tianjin Key Laboratory of Cancer Prevention and Therapy and Tianjin Lung Cancer Center, Tianjin, China; ^2^ Daqing Oilfield General Hospital, Heilongjiang, China; ^3^ Department of Radiotherapy, Peking University 3rd Hospital, Beijing, China; ^4^ Department of Radiation Oncology, Division of Radiation Oncology, The University of Texas MD Anderson Cancer Center, Houston, TX, USA

**Keywords:** brain radiation necrosis, Cyberknife, stereotactic radiotherapy (SRT), biologically equivalent dose (BED)

## Abstract

In this study, we examined the factors contributing to brain radiation necrosis and its predictors of patients treated with Cyberknife radiosurgery. A total of 94 patients with primary or metastatic brain tumours having been treated with Cyberknife radiotherapy from Sep. 2006 to Oct. 2011 were collected and retrospectively analyzed. Skull based tracking was used to deliver radiation to 104 target sites. and the prescribed radiation doses ranged from 1200 to 4500 cGy in 1 to 8 fractions with a 60% to 87% isodose line. Radiation necrosis was confirmed by imaging or pathological examination. Associations between cerebral radiation necrosis and factors including diabetes, cardio-cerebrovascular disease, target volume, isodose line, prescribed dosage, number of fractions, combination with whole brain radiation and biologically equivalent dose (BED) were determined by logistic regression. ROC curves were created to measure the predictive accuracy of influence factors and identify the threshold for brain radiation necrosis. Our results showed that radiation necrosis occurred in 12 targets (11.54%). Brain radiation necrosis was associated by BED, combination with whole brain radiotherapy, and fractions (areas under the ROC curves = 0.892±0.0335, 0.650±0.0717, and 0.712±0.0637 respectively). Among these factors, only BED had the capability to predict brain radiation necrosis, and the threshold dose was 7410 cGy. In conclusion, BED is the most effective predictor of brain radiation necrosis, with a dose of 7410 cGy being identified as the threshold.

## INTRODUCTION

Stereotactic radiotherapy (SRT), including Cyberknife SRT, is used to treat patients with primary and metastatic brain tumors [1–3], whose most common late side effect is brain radiation necrosis[4–6]. Until now, research on tolerated dose of hypofractionated SRT for brain tumors is limited, especially those about the influencing and predicting factors of brain radiation necrosis post Cyberknife radiosurgery. Here we aimed to address this issue by a retrospective analysis and a reference dose for brain tumor patients undergoing Cyberknife therapy was established. Our results also provided advice on how to reduce the risk of brain radiation necrosis.

## RESULTS

### Occurrence of brain radiation necrosis

Brain necrosis occurred in 12 participants (9 males and 3 females) aged 31-70 (median: 54.5). A total of 12 targets were found representing with necrosis with a rate of 11.54%. Among these 12 cases, 1 had primary brain lymphoma, 1 brain metastases from stomach cancer, 1 brain metastases from kidney cancer, 1 brain metastases from small cell lung cancer, 1 brain metastases from esophagus cancer, and 7 brain metastases from non-small cell lung cancer. Four patients had received whole brain radiotherapy, including 3 were prescribed a dose of 30 Gy/10f, and 1 a dose of 46 Gy/23f. For all the targets treated with Cyberknife, the median treatment volume was 5756.91 mm^3^ (2603.68-16250.10 mm^3^), the median radiation dose 2800 cGy (2000-4000 cGy), the median isodose line 77.5% (68-82%), the median number of fractions 2 (1-4), and the median biologically equivalent prescription dose 7920 cGy (6930-13110 cGy; for those who had received whole brain radiotherapy, the value of BED was obtained by summarizing the BEDs in these two treatments). After Cyberknife therapy it took a median of 14 months (5-24 months) for brain radiation necrosis to develop. See Table 1 for details.

**Table 1:** Characteristics of the cases of radiation necrosis

Cases	Age (years old)	Gender	Lesions	Location	Tracing	WBRT	Target volume (mm^3^)	Dose(cGy)	Dose line (%)	Fraction (f)	BED tumor	BED brain	Time of REP after treatment(Months)
Case 1	53	Male	NSCLC	Left parietal occipital	skull tracking	No	9091.04	3600	73	3	7920	25200	7
Case 2	70	Female	NSCLC	Right parietal	skull tracking	No	6616.31	3200	75	2	8320	28800	15
Case 3	31	Female	NSCLC	Right cerebellar	skull tracking	No	2771.79	2300	79	1	7590	28750	12
Case 4	53	Female	NSCLC	Right cerebellar	skull tracking	No	13562.74	3000	77	2	7500	25500	21
Case 5	47	Male	NSCLC	Left occipital lobe	skull tracking	30Gy/10f	4897.5	2000	82	2	7900	19500	10
Case 6	47	Male	SCLC	Left temporal lobe	skull tracking	30Gy/10f	4621.61	2600	77	2	9880	27000	15
Case 7	58	Male	Lymphoma	Left parietal	skull tracking	46Gy/23f	16250.1	2300	82	1	13110	37050	5
Case 8	65	Male	NSCLC	Right parietal	skull tracking	30Gy/10f	2603.68	2300	82	1	12390	36750	24
Case 9	64	Male	Esophageal Cancer	Right parietal	skull tracking	No	11221.52	3600	78	3	7920	25200	14
Case 10	66	Male	NSCLC	Left thalamus	skull tracking	No	7631.72	3300	68	3	6930	21450	11
Case 11	49	Male	Kidney cancer	Brain stem	skull tracking	No	3004.19	4000	74	4	8000	24000	16
Case 12	56	Male	Gastric Cancer	Left cerebellar	skull tracking	No	4194.93	2300	81	1	7590	28750	14

### Factors associating with brain radiation necrosis

The results of the logistic regression showed that combination with whole brain radiotherapy, fractions, and BED were significantly associated with development of brain radiation necrosis in the model, and thus able to contribute to the development. See Table 2 for details.

**Table 2:** Logistic regression analysis for radiation necrosis

Factors	Regression coefficient	Wald value	RR value	95%CI	*P* value
Female vs. male	−0.127	0.01428	0.881	0.109-7.085	0.905
Age	−0.0177	0.239	0.982	0.915-1.055	0.624
Diabetes	0.792	0.327	2.207	0.146-33.282	0.567
Cardio-cerebrovascular disease	−5.178	0.372	0.00564	0.000-94696.156	0.542
Without or with WBRT	−15.764	4.025	0.0000000142	0.000-0.695	0.0448
Target volume	0.00000118	0.030	1.000	0.999-1.001	0.862
Dose	0.0169	0.000316	0.983	0.152-6.342	0.985
Dose line	−0.0463	0.189	0.955	0.775-1.177	0.664
Fraction	−4.075	4.521	0.0170	0.000397-0.727	0.00335
BED	0.00881	5.311	1.009	1.001-1.016	0.0212

### Predictor of brain radiation necrosis

**Figure 1:**
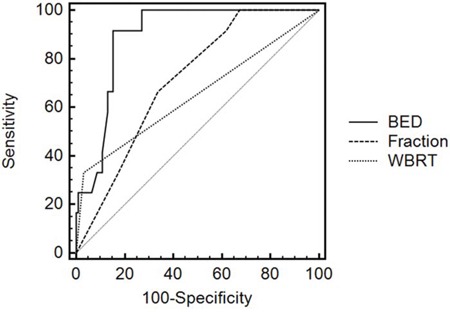
Roc curves of risk factors

MedCalc software was used to create ROC curves, and factors included BED, combination with whole brain radiation, and fractions (Figure 1). The areas under the ROC curves were 0.892±0.0335, 0.650±0.0717, and 0.712±0.0637 respectively, suggesting that BED had more predictive value for brain radiation necrosis than combination with whole brain radiation and fractions. Moreover, the ROC curve analysis showed the BED threshold was 7410 cGy for brain radiation necrosis, and the sensitivity and specificity were well balanced (Table 3). For the sake of clinical practicability, we further calculated the prescription dose in 1-5 fractions on the basis of the threshold values, and recommended dosages to avoid brain radiation necrosis for clinical reference was shown in Table 4.

**Table 3:** Multiple factors in ROC curve analysis

	AUC	95%CI	z statistic	*P* value (Area=0.5)	Associated criterion	Sensitivity	Specificity
With or without WBRT(0:no/1:yes)	0.650±0.0717	0.551-0.741	2.098	0.0359	>0	33.33	96.74
Fraction(f)	0.712±0.0637	0.615-0.797	3.335	0.0009	<=2	66.67	66.30
BED(cGy)	0.892±0.0335	0.816-0.945	11.710	<0.0001	>7410	91.67	84.78

**Table 4:** Recommended dosages for avoiding brain radiation necrosis in clinical practice

Fractions	Dose (Gy/F)
1F	22.68
2F	14.89
3F	11.49
4F	9.44
5F	8.16

## DISCUSSION

Brain radiation necrosis is caused by white matter injury characterized by loss of oligodendrocytes, along with demyelination (Glial Injury Hypothesis) [14, 15] or vasogenic edema (Vascular Injury Hypothesis) [16–18]. Radiation damage to glial cells and vascular endothelial cells can lead to various late effects in the brain, and the extent of the damage depends on the biological dose. This study showed that BED, combination with whole brain radiotherapy, and fractions would contribute to brain radiation necrosis following Cyberknife therapy. BED was higher in patients who had received whole brain radiotherapy as a result of adding together the doses of two treatment regimens. Brain belongs to late-responding tissues, and are found to have low values of α/β ratio, and are more susceptible to a single, high dose of radiation. If the total dose keeps unchanged, fewer fractions will lead to a higher BED, thus increasing the risk of brain necrosis. The ROC curve analysis further confirmed the founding from the logistic regression analysis.

Varying doses are always adopted during Cyberknife therapy for various patients with different treatment plans and treatment volumes. For patients with systemic metastases, palliative care with low-dose radiation was usually given to obtain symptom relief; whereas for patients with a single brain metastasis, a higher dose might have been prescribed. Furthermore, tumours with larger volume usually result in palliative treatment. This study created a good chance for research on radiation tolerance of brain tissue with the various prescription doses, and results showed that the treatment volume did not appear to affect the development of brain radiation necrosis.

The rate of brain radiation necrosis was 11.54%, consistent with previous research [19, 20]. Furthermore, we analyzed the factors affecting and predicting brain radiation necrosis, established the threshold value for brain radiation necrosis and given a recommended prescription dose [21, 22]. More importantly, the study fills a gap in the research on the tolerated dose of hypofractionated SRT for brain tumours, and are also clinically important in terms of avoiding the occurrence of brain radiation necrosis following Cyberknife SRT.

In conclusion, by analyzing the brain necrosis cases, we explored various factors that may affect radiation necrosis in the brain, and the tolerated dose of hypofractionated radiotherapy for brain tumours. A reference dose level has been established to reduce the toxicity of Cyberknife therapy. We believe this study has paved the way for future research that will produce more substantial evidence to prevent brain radiation necrosis following Cyberknife SRT and improve treatment of brain cancer using Cyberknife.

## MATERIALS AND METHODS

### Patient information

The study was carried out in accordance with the institutional ethical guidelines and the use of patient information was approved by the Medical Ethics Committee of Tianjin Medical University Cancer institute and Hospital. Every patient involved in the study was asked to sign a piece of written informed consent which has been approved by the ethics committee of Tianjin Medical University Cancer institute and Hospital. The study was conducted according to the principles expressed in the Declaration of Helsinki. A total of 94 patients (mean age 51.5, range 6-85; 58 males and 36 females) with primary or metastatic brain tumours who had been treated with Cyberknife between September 2006 and October 2011 were collected and analyzed retrospectively. The inclusion criteria included primary or metastatic brain tumours, usage of Cyberknife SRT to treat brain tumours, follow-up of at least two-year, diagnosis of radiation necrosis confirmed by imaging or pathological examination. There were 104 targets, including 81 targets in 81 patients with brain metastases and 23 targets in 13 patients with primary brain tumours. Five patients (7 targets) received whole brain radiotherapy before Cyberknife SRT, among whom 4 (6 targets) were given a dose of 30 Gy/10f; and 1 (1target) 46 Gy/23f. Skull based tracking was used, together with a 60-87% isodose line (median: 79%), a dose of 1200-4500 cGy (median: 2550 cGy) and a BED of 2380-13110 cGy (median: 5130cGy) in 1-8 fractions (median: 3). BED=nd × (1+d/10); n: fraction, d: the dose of one fraction, α/β=10. See Table 5 for details.

**Table 5:** Clinical characteristics of patients

Characteristics	Values
Number of cases (*n*)	94
Number of targets (*n*)	104
Gender	
Male	58
Female	36
Mean age in years (range)	51.5 (6-85)
Targets	
Primary	23
Metastatic	81
Combined with WBRT(Targets)	
Yes	7
No	97
Mean treatment volume in mm^3^ (range)	7805.78 (136.21-92760.70)
Mean dose line in percentage (range)	79 (60-87)
Mean dose in cGy (range)	2550.00 (1200-4500)
Mean fraction in f (range)	3 (1-8)
Mean BED-tumor in cGy (range)	5130 (2380-13110)
Radiation encephalopathy (targets)	
Yes	12
No	92

### Diagnosis of brain radiation necrosis

Comprehensive imaging is the most realistic and most frequently used method in the diagnosis of brain radiation necrosis [7–9]. While pathological examination, although known as the golden standard, was unachievable due to the following reasons. Firstly, many of the brain tumors treated with Cyberknife are located near the skull base or in the important functional areas, resulting in impossibility of surgical resection or stereotactic puncture. Secondly, patients with history of Cyberknife treatment, especially those with multiple lesions, usually rejected a puncture because it is the last choice in clinical practice to perform puncture biopsy of every single lesion. Furthermore, even a stereotactic puncture may not be able to completely display the pathological characteristics of the involved tissue.

Therefore comprehensive imaging is the most realistic and frequently used method in the diagnosis of brain radiation necrosis[7–9], and we chose to make the diagnosis upon patients' medical history, signs and symptoms, along with results of various imaging approaches such as MRI, nuclear magnetic resonance spectroscopy, and PET-CT [10–13] in this study. MRI scan and resonance spectroscopy were conducted first, and PET-CT further introduced if the diagnosis cannot be confirmed. Briefly, most brain necrosis showed irregular shape in MRI with hypointense on T1WI and hyperintense on T2WI. Moreover, liquefaction necrosis often represented with lower signal intensity on T1WI and higher signal intensity on T2WI. After administration of Gd-DTPA, irregularly enhanced signal without enhanced nodular was obtained in the lesions center, while a large area of edema belt in T1 and T2 signal without enhancement around the lesions. In MRS, Cho, Cr and NAA levels were reduced, and NAA/Cho and NAA/Cr radio decreased. In PET, the metabolic rate of brain radiation necrosis was lower than that of normal brain tissue, resulting in decreased uptake of FDG and defected radioactive imaging in the corresponding region. The diagnosis was all ultimately determined by 3 independent investigators. If a patient had severe symptoms but also indications for surgery, the lesion could be resected and the diagnosis confirmed by histology. In this study, there were 12 patients with brain necrosis, with 1 confirmed by pathology and 11 by imaging.

### Follow-up and statistical methods

MRI assessment of brain lesions should be conducted regularly 2 months after the Cyberknife radiosurgery; patients should be re-examined once every 3 months within the following one year, and then re-examined if necessary for a maximum of 6 months. If symptoms of intracranial lesions occurred, re-examination should be done immediately. All statistical analyses were executed using SPSS 17.0 software. The level of significance was defined as *P* < 0.05. Logistic regression was performed to explore associations between brain radiation necrosis and factors including diabetes, cardio-cerebrovascular disease, age, gender, combination with whole brain radiation, prescribed dosage, fractions, isodose line, and BED. MedCalc software was used in ROC curve analysis which included all the statistically significant factors in the logistic regression model, and the threshold values of the factors were estimated.
